# An Efficient Anomalous Sound Detection System for Microcontrollers †

**DOI:** 10.3390/s24237478

**Published:** 2024-11-23

**Authors:** Yi-Cheng Lo, Tsung-Lin Tsai, Chieh-Wen Yang, An-Yeu Wu

**Affiliations:** Graduate Institute of Electronics Engineering, National Taiwan University, Taipei 106319, Taiwan; alan@access.ee.ntu.edu.tw (Y.-C.L.); toryn@access.ee.ntu.edu.tw (T.-L.T.); yolanda@access.ee.ntu.edu.tw (C.-W.Y.)

**Keywords:** anomalous sound detection, Industrial Internet of Things, edge intelligence, model compression, microcontrollers

## Abstract

Anomalous Sound Detection (ASD) systems are pivotal in the Industrial Internet of Things (IIoT). Through the early detection of machines’ anomalies, these systems facilitate proactive maintenance, thereby mitigating potential losses. Although prior studies have improved system accuracy using various advanced machine learning technologies, they frequently neglect the associated substantial computing and storage demands, which are crucial in resource-constrained IIoT environments. In this paper, we propose an ASD system that is efficiently optimized for both software and hardware considerations regarding edge intelligence. For the software aspect, we identify signal variation as a critical issue for ASD. Hence, we introduce a suite of lightweight yet robust processing techniques that enhance accuracy while minimizing resource consumption. As for the hardware aspect, we find that memory constraints may be a significant challenge for deploying ASD systems on microcontrollers (MCUs). Therefore, we propose a memory-aware pruning algorithm specialized for ASD to fit into MCUs’ constraints. Finally, we evaluate our method on the DCASE dataset, and the results show that our system achieves favorable outcomes in both accuracy and resource efficiency, marking our contribution to ASD system practice.

## 1. Introduction

Anomalous Sound Detection (ASD) systems are essential to the Industrial Internet of Things (IIoT) in Industry 4.0 [1]. As the machinery network expands in Industry 4.0, minimizing both downtime and maintenance costs becomes increasingly vital because the opportunity costs of shutdowns and the labor costs of maintenance are substantial. To address this issue, ASD is commonly adopted; by utilizing the IoT and advanced neural network (NN) technologies, ASD systems can promptly detect anomalies by continuously monitoring machine sounds. This early detection enables proactive maintenance, allowing us to perform necessary repairs early to sustain industrial operations. Therefore, by developing effective ASD systems, we can significantly enhance equipment reliability while simultaneously reducing maintenance costs.

**Figure 1:**
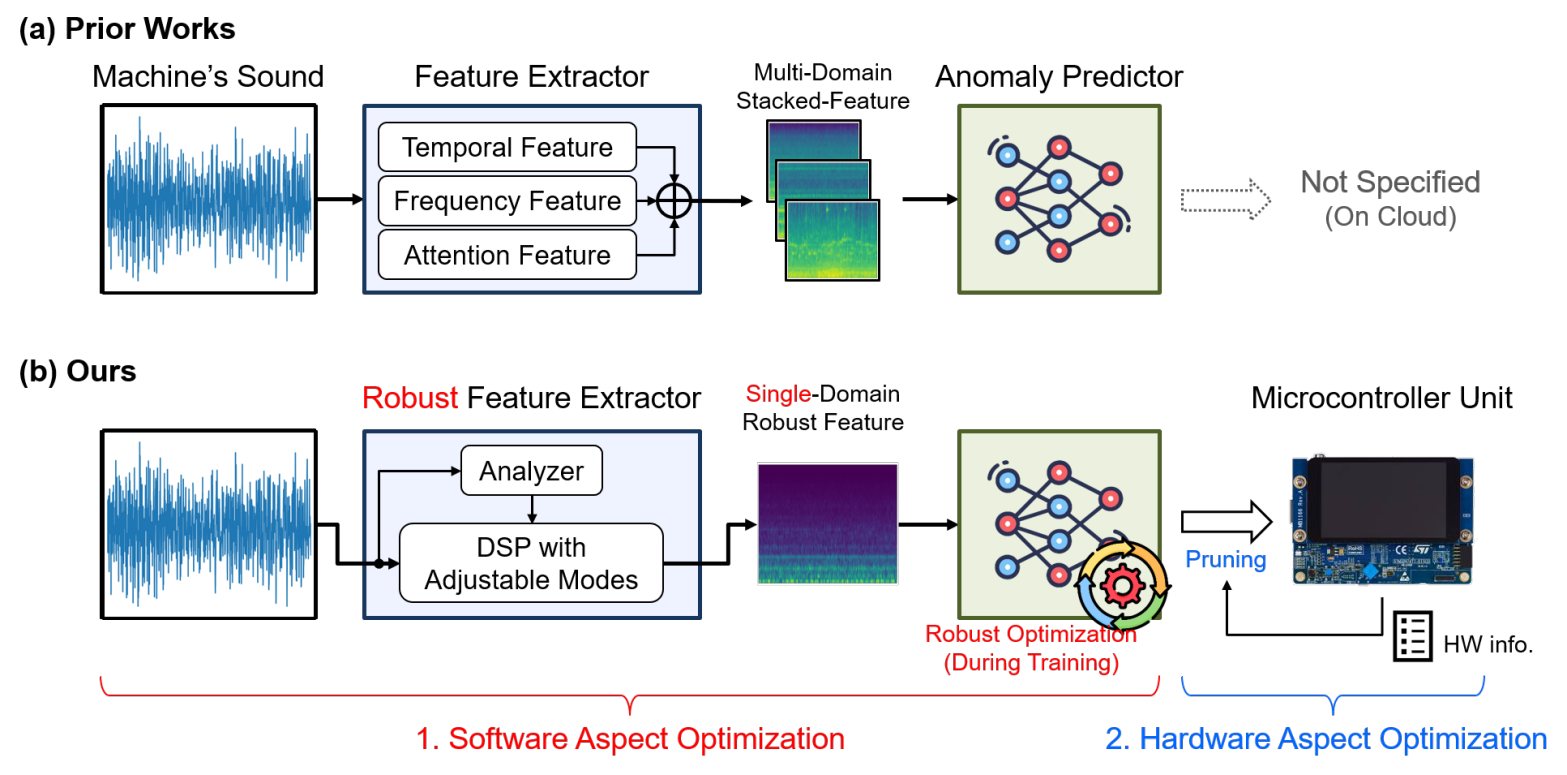
A comparison between prior works and our work. (**a**) Prior works cascade features of multiple domains, and none of them further consider deploying their ASD systems on edge devices. (**b**) Our work applies robust processing, and we also consider system miniaturization regarding the trend of edge intelligence in IIoT.

Related works have primarily focused on software improvement by enlarging the ASD system. These enhancements boost prediction accuracy through various advanced NN techniques, most of which leverage the integration of multiple features from diverse domains [2,3,4,5,6]. Unlike the baseline method that relies on digital signal processing (DSP) calibrated by humans, the adopted NNs can automatically optimize the feature extraction process. Hence, as illustrated in Figure 1a, this approach often involves concatenating independent feature sets from different domains (i.e., time-based, frequency-based, and attention-based). This multi-feature integration can enhance the input information of the subsequent anomaly predictor, thereby facilitating more precise estimations.

However, we contend that these methods may not be entirely practical due to the insufficient consideration of another critical aspect of IIoT [7,8], namely hardware platform deployment. One crucial challenge is that prior approaches often require processing multiple feature domains. This not only significantly increases computing and storage demands, but also escalates the resource requirement of the anomaly predictor, since the input dimension expands. Another challenge lies in deploying ASD systems on edge devices. With the anticipated surge in machine numbers in Industry 4.0, edge intelligence may be a compelling trend. Consequently, it is essential to apply TinyML techniques (e.g., model pruning or quantization) to fit into the resource constraints of edge devices. Unfortunately, previous studies have generally overlooked these considerations.

To address the two primary challenges of system size and edge deployment, we develop a lightweight ASD system and deploy it on a microcontroller (MCU) [9,10]. As illustrated in Figure 1b, our proposed ASD system is end-to-end optimized, including both software and hardware fronts. For the software aspect, we lighten the system by focusing on the core issue of ASD, enhancing the system through robust processing rather than solely relying on NN technologies. As for the hardware aspect, we enable edge deployment by proposing a memory-aware model pruning algorithm to meet MCU constraints while maintaining system accuracy. The main contributions of this paper are as follows:

1. Software aspect optimization—lightweight system by robust processing:We identify that the key issue of the ASD system may be the signal variation caused by the IIoT and ASD scenarios. Therefore, we improve the robustness through the system’s feature extractor, anomaly predictor, and training loss function. The results show that these improvements can significantly boost accuracy without increasing resource demands.
2. Hardware aspect optimization—enabling edge intelligence by pruning ASD for MCUs: We recognize that the critical challenge of deploying the ASD systems on MCUs may be the memory constraints. Thus, we propose a memory-aware pruning algorithm based on reinforcement learning. This algorithm minimizes accuracy degradation caused by model miniaturization while meeting the stringent resource limitations of MCUs.
3. The end-to-end development of an ASD system: We have successfully implemented the full ASD system on an STM32H747I MCU from STMicroelectronics, Italy. This integration of technologies demonstrates the practicality of our software and hardware related optimizations.

The rest of this paper is organized as follows. We illustrate the background and the related works of ASD in Section 2. The software and hardware optimization are introduced in Section 3 and Section 4, respectively. The experimental results on the open data, the DCASE 2020 Challenge Task 2 dataset [1,11,12], are presented in Section 5. Finally, we detail our conclusion in Section 6.

## 2. Background

### 2.1. Preliminary of the ASD Systems

**Figure 2:**
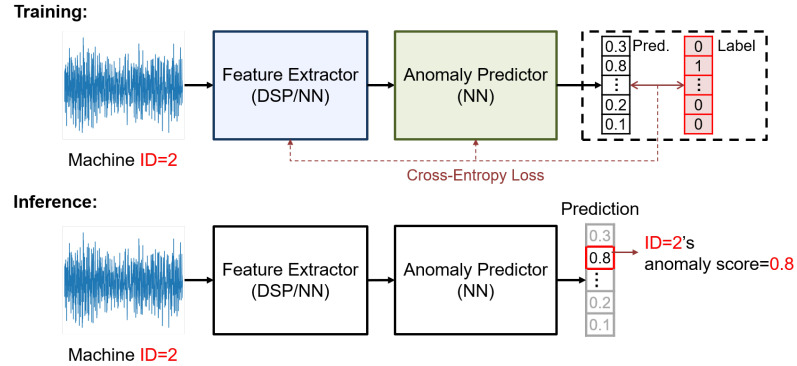
Training and inference flow of discriminative-based ASD systems.

Recent studies on ASD have shown that discriminant-based methods [2,3,4,5,6,13,14,15] outperform traditional generative-based methods [1,16,17,18] in accuracy. Figure 2 illustrates the basic components of such systems, which consist of a feature extractor followed by an anomaly predictor. For the feature extractor, traditional ones utilize DSP to compute the log-Mel spectrogram, an informative time–frequency feature. Recent advances have integrated additional NN-based feature extractors, which can significantly enhance accuracy. As for the anomaly predictor, related works typically employ convolutional neural networks (CNNs) to automatically optimize prediction processes. To this end, the essence of this kind of ASD system is the training and inference of NN to obtain the correct anomaly score.

The training of ASD systems is conducted using self-supervised learning (SSL) due to the scarcity of anomalous data, which precludes direct training on predicting anomaly scores. As a workaround, SSL employs the task of “classifying different machine sound signals” as a pseudo-task. This approach is depicted in Figure 2, utilizing cross-entropy loss $\left(L_{CE}\right)$ as shown below:

(1) $$L_{SSL}=L_{CE}\left(y_{prd},y_{label}\right)\;,$$

where $y_{prd}$ represents the output prediction vector and $y_{label}$ is the one-hot label vector. Through this method, the ASD system learns to recognize the sounds of normal machines. Consequently, when abnormal data are presented, the ASD system would not be able to classify it into any known category, thus identifying it as an anomaly.

During the inference phase, the ASD system evaluates the input data with an anomaly score, where a higher score indicates a higher likelihood of machine failure. A common approach involves using the post-softmax value of the output vector $y_{prd}$:

(2) $$A_{ID}\left(y_{prd}\right)=-\log\left(\;\sigma (y_{prd};\mathrm{ID})+\epsilon \;\right)\;,$$

where σ(·;ID) denotes the ID entry of the post-softmax vector, and ϵ is a small bias term added to avoid undefined results. Since the post-softmax value could reflect the NN’s confidence in each class to a certain extent, this method can indicate how normal the input data are compared to the training data. Finally, if $A_{ID}\left(y_{prd}\right)$ surpasses a predefined threshold, the input data are considered anomalous, triggering actions afterward such as machine maintenance. Notice that the threshold can be adjusted based on specific requirements: set low to sensitively detect potential machine failures, or set high to tolerate minor malfunctions. Consequently, the performance of an ASD system is evaluated by its aggregate accuracy across various threshold settings, simulating different operational scenarios.

### 2.2. Related Works of ASD

In the realm of ASD development, most research primarily concentrates on enhancing system accuracy, and their methods often fall within the scope of enhancing feature extractors by advanced NN technologies. That is, aside from the traditional log-Mel spectrogram, these works incorporate additional NN-based blocks to extract features from multiple domains. For instance, Liu et al. [2] and Guan et al. [3] introduced an extra CNN to capture time-domain features. Since CNN can efficiently capture the time-domain characteristics, these two works enhanced the system’s ability to analyze both on the time domain and the frequency domain. Based on this concept, Chen et al. [4] modified the CNN architecture to access multi-level time-domain information. This allows the system to observe details from different scales. To take a step further, Zhang et al. [5] and Choi et al. [6] implemented self-attention mechanisms on the original architecture. With the ability to capture long-range dependencies and contextual information other than CNNs, ref. [6] achieved state-of-the-art accuracy. To sum up, in contrast to methods that solely rely on log-Mel spectrogram features [13,14,15], the diversified features can significantly enhance accuracy by providing the anomaly predictor with a richer information set.

Despite these advancements, we contend that feature-concatenating approaches might compromise practicality due to their substantial resource requirements. For starters, as the number of machines in Industry 4.0 increases, the bandwidth is not enough for traditional cloud-based systems. This makes deploying ASD systems on edge devices viable, as the edge intelligence can process data locally, reducing data transmission costs. However, existing ASD systems may not be suitable for this scenario because of their high resource demands. Given the limited computing and storage capacities on edge devices, the additional resource burden imposed by multiple features could overwhelm these devices. Therefore, developing a lightweight ASD system rather than using the status quo emerges as a crucial consideration.

## 3. Software Aspect Optimization: Efficient ASD System Design by Robust Processing

In this section, we elucidate our software optimization towards a lightweight ASD system, which solely relies on DSP as the feature extractor. We argue that the critical issue of ASD may be the variation in the signal; therefore, instead of directly applying advanced NN technologies, we take a step back to refine the robustness of the system. Specifically, we enhance the three critical blocks of the system (as in Figure 2): (1) the feature extractor, (2) the anomaly predictor, and (3) the training method. We discuss each block in the following subsections.

### 3.1. Robust Feature Extraction by Dynamic Signal Processing

In ASD systems, signal variation often results from various external noise interferences, which can be mistakenly identified as features of the signal itself. One source of noise originates from IoT sensors, which are naturally susceptible to background noise such as white noise, shot noise, and 60 Hz powerline interference. Additionally, in the complex environments of Industry 4.0, sensors may capture sounds other than those from the targeted machinery, which further contaminates the sound signal. These noises can significantly impair the judgment of the ASD system, leading to decreased accuracy.

**Figure 3:**
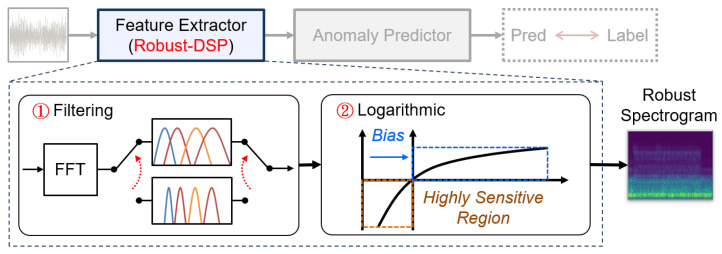
Enhancing the robustness of the feature extractor by dynamic filtering and biasing on the logarithmic operation.

As illustrated in Figure 3, we enhance the DSP module to more robustly process these signals. The adopted DSP operates as a two-stage pipeline: the first stage conducts a Short-Time Fourier Transform (STFT) and applies a Mel filter, while the second stage performs logarithmic operations. Although these processes are effective in standard audio signal processing, they lack adaptability to noise interference and may even exacerbate noise patterns. As prior works also rely on these stages as one of the primary input feature sources, we argue that this is a fundamental reason for the need for additional features to counteract noise effects. To address this challenge, we have improved the robustness of both DSP stages, thereby eliminating the need for feature-stacking from the outset of the system.

Firstly, we enhance the robustness of our filters by dynamically selecting the appropriate filters for the Short-Time Fourier Transform (STFT) results. We suggest that different noise levels require distinct processing approaches, and thus, we alter the filter set based on the signal variance as outlined in [19]. The filtering process is represented as follows:

(3) $$S\left[m,\tau \right]=\sum_{k=0}^{B-1}H\left({var}_{X}\right)\left[m,k\right]\cdot {\left|X\left[k,\tau \right]\right|}^{2}\;,$$

where X and S denote the STFT results and the filtered outcomes, respectively, and $H({var}_{X})$ denotes a set of filters determined by the variance of the signal (${var}_{X}$). The indices *k*, τ, and *m* correspond to the frequency, time, and filter axes, respectively. Notice that the filter sets include the gamma-tone filters and the Mel filters, where the former has a broader window and the latter has a narrower window (refer to the upper and the lower part of Figure 3). This makes these two kinds of filters provide different needs: preserving the information or reducing the noise interference. Hence, by dynamically selecting the applied filters based on the variance, which can represent the noise level to a certain extent, the feature extractor can better adapt to noise.

Secondly, we improve the robustness of the logarithmic operation by finding the proper operating region. This logarithmic operation was originally designed to compress dynamic range and enhance subtle features; however, it becomes unstable under noisy conditions. Since this operation tends to amplify the amplitude of smaller values, it can significantly affect the output under noise interference. To mitigate this issue, we modify the logarithmic function as follows:

(4) $$S_{L}=log\left(\;S+\beta \;\right)\;,$$

where the function’s behavior is illustrated in Figure 3. The tunable biasing term (β) performs as the knob between the sensitivity (lower β value) and robustness (higher β value). Hence, by fine-tuning β, we can ensure the transformed feature ($S_{L}$) remains effective even in noisy environments.

### 3.2. Robust Anomaly Predictor by Structural Learning

After the DSP block, we then optimize the second block, a CNN-based anomaly predictor, to further enhance system performance. We recognize that signal variations due to IoT sensor noise and complex Industry 4.0 environments cannot be completely mitigated by robust DSP techniques alone. Thus, we aim to elevate the robustness of the anomaly predictor to compensate for this insufficiency.

Since CNNs generally perform well with diverse training data, ensuring sufficient variability within the dataset is essential. To address the typically unvaried nature training dataset in ASD, we employ artificial data augmentation techniques. However, it is crucial to balance increasing diversity without distorting the inherent characteristics of the signals, as excessive processing can degrade training effectiveness. In this augmentation, the primary objective is to expand the learning space while maintaining the integrity of the original data.

**Figure 4:**
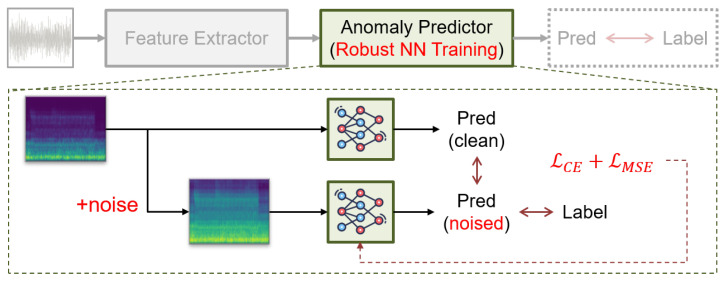
Enhancing the robustness of the anomaly predictor by structural learning.

Inspired by [20], we implement structural learning strategies to manage this balance effectively. As illustrated in Figure 4, this structural learning involves adjusting both data augmentation and loss function. First, for data augmentation, we introduce variability by applying Additive White Gaussian Noise (AWGN) along with some common image-processing techniques. The augmented data can be represented as follows:

(5) $$S_{L,n}=S_{L}+N\left(0,{\tilde{\sigma }}^{2}I_{S_{L}}\right),\;\mathrm{where}\;\tilde{\sigma }=\frac{amp(S_{L})}{10^{\mathrm{PSNR}/20}}.$$

Here, $N(0,{\tilde{\sigma }}^{2}I)$ denotes the multivariate normal distribution with mean zero and variance ${\tilde{\sigma }}^{2}$, with PSNR being the adjustable parameter that controls augmentation level based on empirical rules. Second, for the loss function, we modify it by incorporating the concept of group lasso. Based on the original function in Equation (1), we improve its efficiency by adding guidance:

(6) $$\begin{array}{cc} \; & L_{all}=L_{SSL}+\gamma \cdot L_{Guide},\;\mathrm{where}\\ \; & L_{SSL}=L_{CE}\left(\phi \left(S_{L,n}\right),l_{class}\right)\;\mathrm{and}\;L_{Guide}={∥f-f_{n}∥}_{F}\;. \end{array}$$

ϕ(·) denotes the CNN-based anomaly predictor, and $∥f-f_{n}{∥}_{F}$ in the lasso for guidance represent the Frobenius norm on the difference of the hyper-features between the normal data and the augmented data.

This structural learning involves forwarding both normal and augmented data simultaneously. Then, during back-propagation, the CNN’s parameters are also simultaneously updated by both data: the augmented data serve as dynamic data, while normal data provide a baseline for the hyper-features. This allows the network to learn from simulated variability while being anchored by the fundamental characteristics of the data, ensuring the CNN combines ergodicity and learning efficiency.

### 3.3. Robust SSL Training Strategy by Reformulation of Objectives

In this part, we focus on optimizing the final block of the ASD system, specifically the training formulation. Given the abundance of normal machine sounds and the scarcity of anomalous data in the ASD training dataset, self-supervised learning (SSL) has emerged as a commonly used and effective alternative. Previous methods typically used classifying sounds from different machines as a pseudo-task. However, we found that this approach might lead to overfitting in the CNN-based anomaly predictor and fail to capture crucial information. This issue often arises because the unvaried nature of the ASD training datasets makes them easy to learn, as they represent only partial machine conditions and not the full spectrum. Consequently, the CNN may develop biases towards specific, non-representative data characteristics.

**Figure 5:**
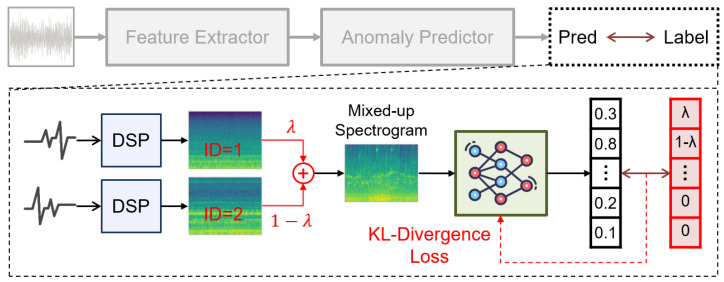
Addressing the over-fitting issue by reformulation of the self-supervised learning (SSL).

To address this issue, we reformulate the training objective of SSL to focus on component prediction, thereby enhanScing robustness. A critical challenge in the original SSL approach is the simplicity of its pseudo-tasks. Thus, inspired by [6,21], we employ the mix-up technique that randomly merges data from any two machines to increase task complexity. The process is illustrated in Figure 5, where we show that the main modification is the alteration in the training task. This way, the anomaly predictor not only has to identify but also distinguish the blended characteristics of different machines.

Similar to what we demonstrated in structural learning, this component-predicting objective also includes generating data and revising the loss function. First, for data generation, we mix up the features of the two distinct machines ($S_{L,i}$ and $S_{L,j}$) using the following formulation:

(7) $${\hat{S}}_{L}=\lambda \cdot S_{L,i}+\left(1-\lambda \right)\cdot S_{L,j}\;\mathrm{with}\;i\neq j\;,$$

where *i* and *j* are indices representing the *i*th and *j*th machine, and λ is coefficient for blending. Notice that *i*, *j*, and λ are random variables, where *i* and *j* follow the uniform distribution whereas λ follows a beta distribution (λ∼Beta(0.5,0.5)). To this end, the training dataset of the ASD system becomes $\left\{{\hat{S}}_{L}\right\}$, with the pseudo-task evolving to predict *i*, *j*, and their respective mixing proportions λ and (1−λ). Second, concerning the loss function, we simplify and generalize it to predict the entire probability distribution of each machine’s proportion in the mix. The label is formulated as follows:

(8) $${\hat{y}}_{label}=\lambda \cdot h_{OH}\left(i\right)+\left(1-\lambda \right)\cdot h_{OH}\left(j\right)\;,$$

where $h_{OH}\left(i\right)$ denote the one-hot encoding on the *i*th entry. Accordingly, the loss function for the SSL pseudo-task in Equations (1) and (6) can be updated as follows:

(9) $$L_{SSL}=L_{KL}(\;{\hat{y}}_{prd}\;||\;{\hat{y}}_{label}\;)\;,\;\mathrm{where}\;{\hat{y}}_{prd}=log\left[\;\sigma \left(y_{prd}\right)+\epsilon \;\right]\;.$$

Here, σ(·) denotes the softmax function, and $L_{KL}\left(\;\cdot \;||\;\cdot \;\right)$ denotes the Kullback–Leibler divergence loss, an efficient evaluator to compare two distributions.

## 4. Hardware Aspect Optimization: Deployment of ASD System on MCU

In this section, we elucidate our hardware aspect optimization in order to deploy our lightweight ASD system on an MCU platform to realize edge intelligence. As the main challenges are the storage constraints of flash and SRAM, we perform memory-aware structural pruning on the CNN part of the ASD system to reduce the storage cost. Through our method, we can completely maintain the system’s accuracy while meeting the constraints. Afterward, we deploy the miniaturized system on an MCU platform as a proof of concept using some open-source tools.

### 4.1. Basics of Deployments on MCUs

**Figure 6:**
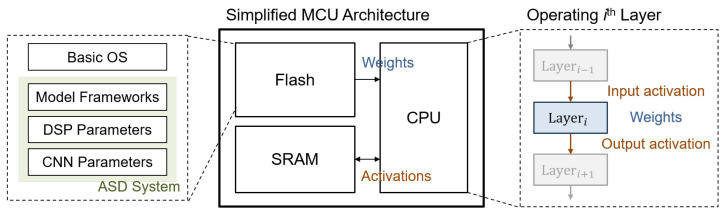
Simplified operating flow of MCU.

In this paper, we explore the use of MCUs as edge devices, detailing their numerous advantages. First, MCUs are cost-effective and energy-efficient, making them well-suited for budget-conscious industrial environments. Second, their compact size enables seamless integration into a variety of industrial machinery. Third, the versatility of MCUs supports a broad range of peripherals and interfaces, accommodating diverse applications. Lastly, they are capable of real-time processing, which is essential for time-sensitive monitoring tasks. The simplified operating flow of an MCU is depicted in Figure 6, illustrating the sequential operation of each block by the storage and computing units.

To this end, deploying an ASD system on an MCU may introduce constraints on storage capacity, specifically flash and static random-access memory (SRAM) [22]. Flash memory stores fixed parameters such as NN weights, while SRAM handles dynamic parameters like extracted features and NN activations. For the flash constraint, the total size of the system’s static parameters must not exceed the available flash capacity. For the SRAM constraint, as the system is operated layer by layer, as shown in Figure 6, the size of dynamic intermediates processed by each layer must fit within the SRAM’s capacity. These constraints are critical for the successful deployment of an ASD system on an MCU, as violating either constraint may result in being unable to operate. Thus, the system requires further optimization to strictly satisfy the constraint to ensure functionality.

To meet these constraints, TinyML technologies [23] are often employed to minimize the system’s resource overhead, particularly the NN component. Among them, neural network pruning [24] is a widely used technique, which involves removing less important parameters. Although this method leverages the inherent sparsity of NNs to significantly reduce complexity, it typically involves a minor trade-off in accuracy. Consequently, numerous prior studies have focused on developing innovative algorithms that significantly reduce the size of the NN while maintaining its accuracy. In this paper, we adopt AMC [25] as our baseline pruning algorithm for its well-proven effectiveness.

### 4.2. Memory-Aware Model Pruning by Reinforcement Learning

In this paper, we implement structural pruning to meet the constraints of our target MCU device. To genuinely benefit the storage overhead, our structural pruning removes entire filters (along the output channel axis) once they are identified as unimportant. To this end, the estimation of a filter’s importance is a critical issue, which should be determined by its impact on both the accuracy of the anomaly predictor and the resource costs. Due to the complexity of this estimation, we employ reinforcement learning (RL) as in [25] to automatically optimize the process.

**Figure 7:**
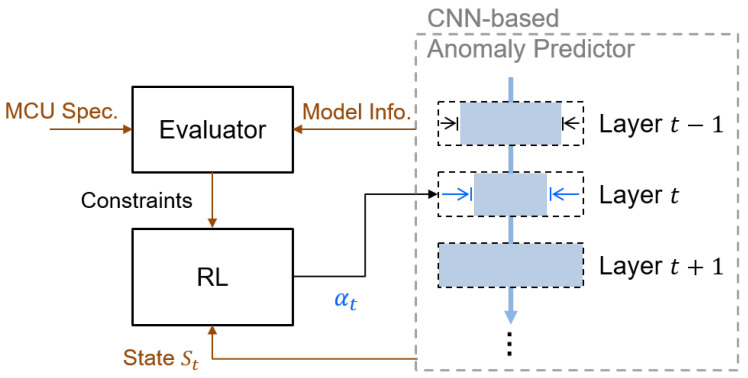
Illustration of our reinforcement learning (RL)-based structural pruning. Notice that in addition to the CNN’s information, the RL also takes the constraints of MCU into consideration.

Figure 7 depicts our pruning strategy, where the RL is the core to assess filters’ importance. This estimation is carried out on a local and global scale. Locally, within the same layer, importance is measured by the aggregate magnitude of a filter’s weights. Globally, across the entire NN model, the RL agent assigns a sparsity ratio to each layer based on its importance: the more crucial the layer, the higher its sparsity ratio, and vice versa. Therefore, the pruning strategy achieves substantial gains by considering both scales: locally, it removes filters with the least impact to minimize their effect on the output; globally, it uses −error×log(flops) as a reward function to evaluate the impact of the pruning sequence.

The design of RL is based on the AMC design [25], and we adapt the state space and agent for our specific requirements. First, the state space ($S_{t}$) is defined as follows:

(10) $$S_{t}=\left(t,c_{i},c_{o},k,w,s,Reduces,Rest,\alpha_{t-1}\right)\;,$$

where *t*, $c_{i}$, $c_{o}$, *k*, *w*, and *s* denote the layer number, input channel, output channel, kernel size, weight size, and stride number of the *t*th layer of the CNN model, respectively. Reduces denotes the reduced parameter count before the *t*th layer, and Rest denotes the remaining parameter count after the *t*th layer. $a_{t-1}$ denotes the estimated sparsity of the (t−1)th layer, which is within a continuous space $a_{i}\in \left(0,1\right]$. Second, the agent then provides an estimated sparsity for the *t*th layer based on the state space. Notice that we incorporate storage constraints to ensure the CNN model meets the overall requirements. As the peak memory usage governs local intermediate storage, the targeted layer sparsity is adjusted accordingly:

(11) $$\alpha_{t}=\min\left(\;\alpha_{peak,t}\;,\;\alpha_{prd,t}\;\right)\;,$$

where $\alpha_{peak,t}$ is the computed bound of sparsity, and $\alpha_{prd,t}$ is the predicted sparsity from the agent. Finally, the process then progresses to the next layer ((t+1)th layer) and repeats, thus systematically miniaturizing the entire model within the constraints. The complete pruning process is summarized in Algorithm 1.
**Algorithm 1** Targeted sparsity of each layer regarding resource constraints1: Compute the initial sparsity ($\alpha_{param,t}$) by [25]2: Compute the sparsity bounded by SRAM ($\alpha_{peak,t}$) through Equation (13)3: Compute the sparsity predicted by the agent: $\alpha_{prd,t}\leftarrow \mu^{\prime }\left(S_{t}\right)$4: Finalize the sparsity for layer *t*: $\alpha_{t}\leftarrow \min\left(\;\alpha_{param,t}\;,\;\alpha_{peak,t}\;,\;\alpha_{prd,t}\;\right)$5: **return** 
$\alpha_{t}$


### 4.3. Estimated of Resource Utilization

A key challenge in the RL process is accurately estimating the performance of the pruned model, particularly in terms of resource utilization. During each iteration, RL needs to assess the strategy’s effectiveness by verifying whether the system remains within the constraints of the MCU. The direct method is to deploy the system on the MCU to measure actual costs; however, this is immensely time-consuming and can significantly extend development timelines. Therefore, we employ an analytical approach to estimate resource utilization, particularly focusing on flash and SRAM constraints.

For the flash constraint, the estimation involves calculating the parameter size of the entire system. As demonstrated in Figure 6, all static parameters are stored in flash memory, including the background operating system and the ASD system itself. As the prunable parameters only exit on the CNN-based anomaly predictor, the relevant constraint equation is formulated as follows:

(12) $$\sum_{i}∥W_{i}∥\leq ∥\mathrm{flash}∥-∥\mathrm{OS}∥-∥\mathrm{ASD}\;\mathrm{System}\;\mathrm{Op}∥-∥\mathrm{DSP}∥\;,$$

where $W_{i}$ denotes the weights of *i*th layer of the CNN, and ∥·∥ denotes the estimated size on flash. Given that pruning only affects the CNN channels’ size and the system architecture remains unchanged, the right side of the equation can be considered a constant.

As for the SRAM constraint, the estimation includes all necessary intermediates, which depend on the CNN’s execution sequence and architecture design. For the execution, while many works have demonstrated significant resource reduction by optimizing the compiler, it is out of the scope of this work. Instead, we adopt the open-source compiler to deploy the ASD system on the MCU board, which operates the system layer by layer for coarse-grained analysis. Next, given that the residual structure is in many adopted CNN structures (e.g., ResNet [26], MobileNetV2 [27], MobileFaceNet [28]), the parallel path should be taken into consideration during the estimation. As we adopt the MobileFaceNet as the backbone model, the finalized formulate can be obtained as follows:

(13a) $$\alpha_{wall1}=\frac{∥\mathrm{SRAM}∥-∥\mathrm{Residual}\;\mathrm{Path}∥-h_{t}\times w_{t}\times c_{i,t}}{h_{t}\times w_{t}\times c_{o,t}}\;,$$

(13b) $$\alpha_{wall2}=\frac{∥\mathrm{SRAM}∥-∥\mathrm{Residual}\;\mathrm{Path}∥}{2\times h_{t}\times w_{t}\times c_{o,t}}\;,\;\mathrm{and}$$

(13c) $$\alpha_{\mathrm{peak},t}=\min\left(a_{wall1},a_{wall2}\right)\;.$$

**Figure 8:**
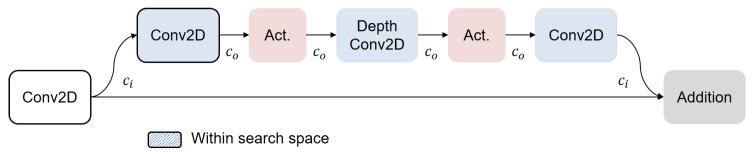
An illustration of the residual block in MobileFaceNet [28].

These equations account for the storage of both input and output intermediates of a layer, along with the SRAM space occupied by the residual path. By this comprehensive analysis, we can derive the appropriate sparsity ratio for each targeted layer as depicted in Figure 8.

## 5. Experiments

### 5.1. Experimental Setup

To evaluate our method, we validate our ASD system on the public DCASE 2020 Challenge Task 2 dataset [1,11,12]. The dataset consists of sound data from 6 different types of machines (fan, pump, slide, valve, toy car, and toy conveyor), and each type has a different number of machine individuals (a total of 41 distinct machines). Notice that every machine only has normal data for training, and the dataset does not have any anomaly data for training. Based on our setup, we regard each single machine as an independent class in our anomaly predictor.

Regarding the system architecture, we adopt our robust DSP and anomaly predictor. For the DSP, the FFT has a window size of 1024, and the temporal sliding window has a hop length of 512. The extracted feature is then undergone through either Mel filters or gamma-tone filters, each of which has 128 bins. As for the anomaly predictor, we employ MobileFaceNet [28] as our backbone network due to its well-proven lightweight merit and high accuracy in related ASD works [2,3,5]. As our focus is not on modifying the CNN architecture, this choice provides a solid benchmark for comparison within our research. The setup for training can be found in Table 1.

As for the targeting MCU, we select STM32H747I as our platform [29], as it satisfies our need for operating NN models and is also suitable for IIoT applications. For computing, this MCU contains dual cores, a Cortex-M7 and a Cortex-M4 core, enabling floating point operations. As for the storage, this MCU includes 2 MB of Flash memory and 1 MB of RAM. Notice that although the corresponding development kit is also provided by STMicroelectronics, we use our own deploying method customized for our ASD system.

Finally, we evaluate our work through the two aspects of ASD in IIoT: accuracy and resource overhead. These comparisons can be found in prior works, including some single-feature approaches [13,14,15] and some multiple-feature approaches [2,3,4,5,6]. Notice that the accuracy is measured by area under curve (AUC) and partial AUC (pAUC), since the conventional accuracy may be influenced by the selection of the threshold value. Following the setting of prior works [2,3,4,5,6,13,14,15], we set the region of interest of the pAUC at 0.1.

### 5.2. Experimental Results of Software Aspect Optimization

We first evaluate the software-aspect optimization in terms of accuracy and resource overhead. Since this phase of testing is independent of the hardware platform, we compute the total number of floating-point operations (FLOPs) to represent computing costs and total parameter numbers to measure storage overhead.

Table 2 presents the AUC of our method compared to others, where we distinguish them between single-feature and multi-feature approaches. Notably, our method consistently outperforms single-feature methods, showing average improvements of 2.78% in AUC and 3.18% in pAUC. In addition, it also surpasses multi-feature methods in many cases, achieving average increases of 1.34% in AUC and 1.57% in pAUC over the leading method [5]. These results suggest that time-frequency features may provide sufficient information and that our robust design adjustments for IoT and ASD non-idealities are more effective than concatenating features from multiple domains.

Table 3 details our advancements in pAUC across our three techniques (robust DSP, robust NN, and robust SSL) compared to other single-feature studies. The results show that each enhancement performs comparably to the state-of-the-art [15]. However, the results also show that each technique also exhibits specific weaknesses across different machine types. This implies that a universal solution is elusive, which highlights the necessity of end-to-end improvements to address all potential complex scenarios of ASD comprehensively.

Table 4 outlines the theoretical resource costs of various methods, where we also categorize them by single-feature and multi-feature variants. It should be noticed that the Baseline [13] represents the results provided by DCASE, and we choose S-gram [2] as our backbone for its effectiveness of the MobileFaceNet. From the table, we can observe that concatenating multiple features significantly increases both computing and storage demands during feature extraction. Conversely, our method introduces a dynamic feature extractor, which only results in about 0.1% in additional FLOP costs. Since the main architecture remains unchanged, the incremental overhead is virtually negligible. Thus, compared to the state-of-the-art method [5], our approach achieves a 16.9% reduction in FLOPs and a 43.5% decrease in parameter counts.

**Figure 9:**
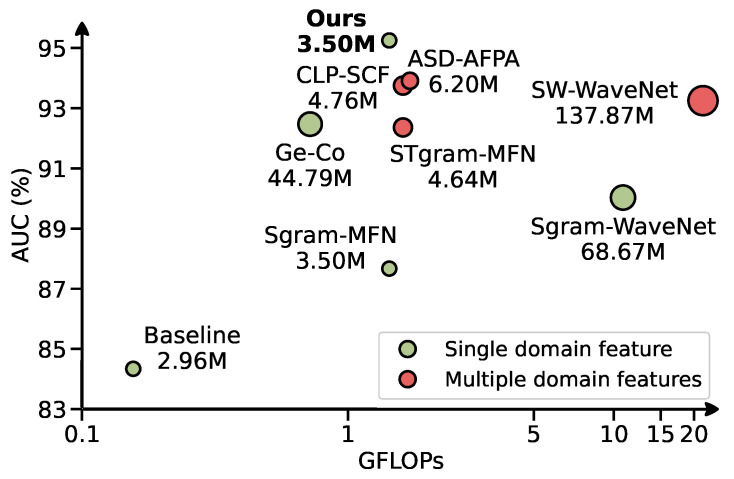
The channel sparsity of the pruned CNN model with ratio equals 0.5.

To further demonstrate our contributions, we depict the AUC versus resource cost in Figure 9. The figure illustrates that concatenating features indeed improves accuracy: the highest AUC achievable with a single feature (Ge-Co [15]) matches only the lowest AUC observed with multiple features. This result underscores the limitations of using the conventional Mel spectrogram alone. However, limited improvement can be obtained through adjusting the feature types or the structure of the anomaly. In contrast, our approach exhibits a superior balance between AUC and resource costs. Given that the only modification involves enhancing robustness without altering the system’s architecture, these results affirm the efficacy of our strategy in improving robustness.

### 5.3. Experimental Results of Hardware Aspect Optimization

After evaluating the software aspect of our optimization, we proceeded to analyze the hardware implications. Table 5 presents the outcomes of our structural pruning compared with the baseline method, AMC [25]. Notice that the *Ratio* represents the sparsity of the entire CNN-based anomaly predictor, indicating the proportion of parameters retained compared to the original model. For the baseline, the primary challenge was satisfying to the peak SRAM memory usage. Lacking direct knowledge of the SRAM constraint, it resorted to aggressive pruning to meet these limitations (a sparsity of only 0.15). Conversely, our method effectively meets both flash and SRAM constraints with a more moderate sparsity ratio of 0.5. The results demonstrate that on the STM32H747I MCU platform, our approach not only complies with hardware limits but also enhances AUC by 1.59% over the AMC method.

**Figure 10:**
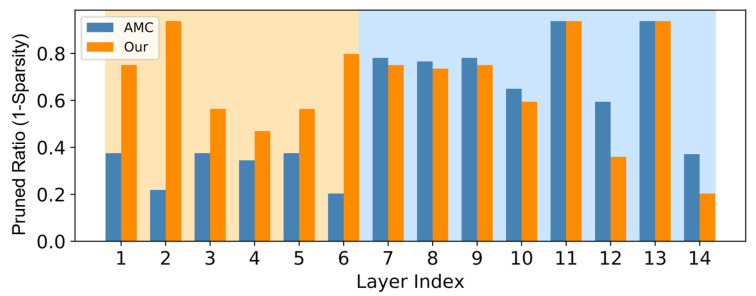
Channel-wise results of the pruned CNN model with ratio equals 0.5.

We further illustrate the impact of our algorithm in Figure 10, which demonstrates the pruned portions of each layer in the CNN model. Notably, the initial layers have lower flash requirements but higher SRAM costs, while the latter layers present the opposite characteristics. Without explicit constraints, AMC tends to target the latter layers due to their inherent higher sparsity. However, this approach risks exceeding SRAM limits during execution. In contrast, our method strategically prioritizes the pruning of the earlier layers, and preserves the latter layers to maintain accuracy. As indicated in Table 5, although our method might slightly reduce accuracy under the same pruning ratio, this strategy ensures compliance with hardware constraints and ultimately results in improved performance on the device.

Finally, we deploy the entire ASD system on an STM32H747I MCU from STMicroelectronics, Italy using some tools, to validate our approach. First, we converted the model into the standard TFLite format using the onnx2tf [30] open-source tool, which automatically reorders the weights to minimize on-device computational overhead. After that, we transformed the TFLite model into binary machine code suitable for the MCU platform. As our system contains both DSP and NN components, we utilize ARM’s CMSIS-DSP and CMSIS-NN libraries to streamline and optimize the compilation process. Ultimately, the main CNN component of our system processes data in approximately 1302 ms, well below the duration of a single 10-s sound data segment. This performance underscores the practicality and effectiveness of our ASD system, representing a significant advancement towards real-world applications in Industry 4.0.

## 6. Conclusions

In this paper, we present a comprehensive approach to enhancing not only the accuracy but also the efficiency of ASD systems deployed on MCUs within the IIoT applications. Our method includes both the software aspect and the hardware aspect. For the software part, we focus on enhancing the robustness of the ASD system instead of concatenating features as in prior works. This significantly enhances system accuracy while avoiding escalating resource costs. As for the hardware part, we introduce an RL-based memory-aware pruning to fit into the MCU’s storage constraints. Our methodologies not only ensure the preservation of accuracy but also significantly reduce the resource overhead, making the implementation more viable in real-world industrial applications. The analytical and experimental results confirm the practicality of our approach, highlighting its potential to facilitate more robust and scalable ASD solutions in the era of Industry 4.0.

## 7. Future Work

Building on the achievement of our work, we recognize that there are opportunities for further software and hardware enhancements, particularly in domain adaptation. A primary challenge is the data domain shifting, which requires the adaptability of the ASD system. In IIoT scenarios, where time and resources are limited for training on data from new machines, ensuring the ASD system’s generalization to unseen data is crucial. Moreover, as the system leverages edge intelligence, optimizing the hardware for on-device adaptation remains an area ripe for exploration. We encourage researchers interested in these challenges to advance this important field.

## Figures and Tables

**Table 1:** Parameters Setting.

Robust DSP	
Mel-spectrogram setup	Bin #: 128, frequency: [0, 8000]
Gamma-tone setup	Ear factor: 27.8, min bandwidth: 24.7, order: 1, frequency: [0, 8000]
Logarithmic setup	Bias: 1
Robust NN	
Training setup	PSNR: 25, regularization λ: 1

**Table 2:** AUC of different ASD approaches.

Method	Fan	Pump	Slide	Valve	Toycar	ToyCon.	Avg.
– Multiple-feature							
STgram-MFN [2]	94.04	91.94	99.55	99.64	94.44	74.57	92.36
CLP-SCF [3]	96.98	94.97	99.57	99.89	95.85	75.21	93.75
SW-WaveNet [4]	97.53	87.27	98.96	99.01	95.49	81.20	93.25
ASD-AFPA [5]	97.55	94.46	99.69	99.12	96.15	76.49	93.91
TASTgram [6]	**98.32**	95.44	**99.53**	**99.95**	96.76	77.90	94.65
– Single-feature							
Sgram-MFN [2]	82.36	87.74	99.08	89.91	88.73	78.17	87.67
S-WaveNet [4]	83.45	85.94	98.00	97.68	94.54	80.53	90.03
Ge-Co [15]	92.73	93.09	98.61	99.06	96.62	74.69	92.47
Ours	93.96	**96.05**	99.08	99.91	**97.13**	**85.37**	**95.25**

The underlined values denote the best performance in single/multiple domain feature, respectively; the **bold values** denote the best performance among all methods.

**Table 3:** The partial AUC (pAUC) of other ASD approaches using a single domain feature.

Method	Fan	Pump	Slide	Valve	Toycar	ToyCon.	Avg.
Baseline [13]	74.40	76.50	85.22	87.98	85.92	56.43	77.74
Glow-Aff [14]	65.30	73.80	82.80	75.00	84.10	59.00	73.90
Sgram-MFN [2]	53.75	67.62	**98.07**	65.88	66.32	67.79	69.91
Ge-Co [15]	85.19	86.89	95.26	95.52	89.33	65.82	86.34
Ours (DSP)	83.29	82.61	97.10	97.40	89.02	67.10	86.10
Ours (NN)	83.44	85.95	96.28	97.59	89.41	61.30	85.66
Ours (SSL)	86.91	89.16	95.89	**99.96**	92.11	67.29	88.55
Ours	**87.57**	**89.31**	95.38	99.54	**92.14**	**73.21**	**89.52**

The **bold values** denote the best performance among all methods.

**Table 4:** Number of floating-point operations (FLOPs) and parameter for each part of different ASD systems.

Method	Feature Extraction		Anomaly Predictor	
	**FLOP**	**Param. #**	**FLOP**	**Param. #**
– Multiple-feature				
STgram-MFN [2]	215 M	1.12 M	1.40 G	3.52 M
CLP-SCF [3]	215 M	1.12 M	1.40 G	3.64 M
SW-WaveNet [4]	74 M	0.52 M	21.56 G	137.35 M
ASD-AFPA [5]	311 M	2.68 M	1.40 G	3.52 M
– Single-feature				
Baseline [13]	41 M	<1 K	0.16 G	2.96 M
Sgram-MFN [2]	41 M	<1 K	1.38 G	3.50 M
S-WaveNet [4]	41 M	<1 K	10.78 G	68.67 M
Ge-Co [15]	41 M	<1K	0.72 G	44.96 M
Ours	41 M	<1 K	1.38 G	3.50 M

**Table 5:** Performance of model pruning for ASD on the MCU.

Method	Ratio	AUC	Parameter # (Cap: 0.488M)	Peak Memory (Cap: 0.5M)	Deployable
Full model	–	95.25	0.875 M	2.197 M	X
AMC [25]	0.5	94.80	0.437 M	2.012 M	X
	0.3	94.41	0.267 M	1.475 M	X
	0.15	93.18	0.134 M	0.485 M	O
Ours	0.5	94.77	0.437 M	0.485 M	O

## Data Availability

The raw data supporting the conclusions of this article will be made available by the authors on request.
